# Older people’s attitudes towards emerging technologies: A systematic literature review

**DOI:** 10.1177/09636625231171677

**Published:** 2023-05-19

**Authors:** Mengxi Zhang

**Affiliations:** University College London, UK

**Keywords:** attitudes, literature review, older people, technology

## Abstract

Public attitudes towards technology have been studied extensively for decades, but older people were not largely involved in early studies. In recent years, with the trend of digitalisation and the rapid growth of the older population around the world, the attitudes of older people towards emerging technologies have attracted the attention of researchers. This article is a systematic review of 83 relevant studies, to summarise the factors that impact older adults’ attitudes towards adopting and using technology. It is found that older people’s attitudes are influenced by their personal characteristics, technology-related factors and the social context of technology adoption. The complex relationship between older people and technology is interpreted by researchers with the framing of older people’s identity, the role of technology, the interaction of the above factors and the opportunity for older adults to act as co-designers.

## 1. Introduction

The relationship between the public and technology is a common topic in Science, Technology and Society (STS) research. In recent years, the trend of global digitalisation and rapid development of technology have inevitably created new interactions and power relations. The notion of users has also been extended in this context, as existing research shows that users are not only appropriating technology under fixed configuration, but further emphasises the concept of people as innovators and contributors (e.g. Lüthje et al., 2005). According to the idiom of ‘co-production’, digital technologies are the products as well as the components of the society, in which the development of technology and social supports are inseparably interconnected (Jasanoff, 2004: 2).

There has been an abundance of literature in various fields investigating the factors that affect people’s perceptions of and attitudes towards new technology. Research on demography, education and communication argues that the attitudes are related to people’s own characteristics, such as gender (Cai et al., 2017), age (Olson et al., 2011) and education (Riddell and Song, 2017). Research on technology studies tends to attribute the reasons for these attitudes to factors of the technology itself, such as cost, ease of use and usability of technology, most of which are incorporated into models like the technology acceptance model (TAM) (Davis, 1989; Venkatesh and Davis, 2000) and the unified theory of acceptance and use of technology (UTAUT) (Venkatesh et al., 2003). STS studies consider broad organisational and social factors as the context in which people interact with technology, including social networks (Bandiera and Rasul, 2006), conflicts of interest among different stakeholders (Elmustapha et al., 2018; Eubanks, 2007), and ethics, values and politics embedded in technology (Miller et al., 2021).

It is found that most of these studies are based in the workplace or organisational settings, with a relative lack of older populations (Peek et al., 2016). As many countries are experiencing rapid growth in the ageing population in the twenty-first century, discussions about how technologies are shaping and being shaped by the ageing society become increasingly important. Research on the relationship between technology and an ageing society, on the one hand, points out the impact of technologies on older people, such as how older people use assistive technology to remain independent and care for themselves (Loe, 2010) and use communication technology to build interactions (Czaja, 2017); on the other hand, it describes new concepts of technology endowed by older adults by observing older people’s attitudes (e.g. Demiris et al., 2004; Mitzner et al., 2010) and engagement (Fischer et al., 2020) in technology. Moreira (2016) encapsulates a ‘wave model’ of how older people, technology and the social system interact with each other, indicating that technological innovation is characterised by different phases related to social and economic organisation and the changing socio-technical conditions can also affect the way people grow old.

Based on the above background, this article develops a systematic review of how older people think of and are affected by technology. The main research questions are (1) What are the factors that impact the attitudes of older adults in adopting and using technology? (2) How are these factors constructed and framed by researchers? This review aims to provide a comprehensive understanding of how older adults interact with new technologies and contribute insights into the future of technology deployment in an ageing society.

## 2. Methods

### Search strategy

A systematic review is best-known for systematically searching, evaluating and synthesising research evidence to bring together existing knowledge in an identified area (Grant and Booth, 2009). This systematic literature review incorporated studies from three key databases: SCOPUS, Web of Science and ProQuest to include extensive literature from natural science, social science, and technology studies as well as cross-disciplinary literature in various contexts. The inclusion criteria of literature were (1) all participants of the research were older people; (2) empirical studies focusing on older people’s attitudes and perceptions of digital technology (those focusing on older technology are not included), or their opinions before and after adoption; (3) published in English in academic journals.

The search items were: (‘older people’ OR ‘older adults’ OR elder*) AND technology AND (attitude OR acceptance OR adoption). In the case of sorting by relevance, the outcomes came to be less relevant around the tenth page (i.e. the first 200–250 articles in each database), and the search ended there. The original search was conducted in May 2022 and 558 articles were identified. After removing duplicate articles and scanning abstracts, a total of 104 papers were obtained. In the next step, the author further screened out 21 articles through full-text assessment, as articles only about theoretical discussions and not only involving older participants were excluded. The final sample for review contains 83 research articles (Table 1).

**Table 1.** References for studies included in this literature review.

No.	Reference
1	Ahn M, Beamish JO and Goss RC (2008) Understanding older adults’ attitudes and adoption of residential technologies. *Family and Consumer Sciences Research Journal* 36(3): 243–260.
2	Arber S, Vandrevala T, Daly T and Hampson S (2008) Understanding gender differences in older people’s attitudes towards life-prolonging medical technologies. *Journal of Aging Studies* 22(4): 366–375.
3	Askari M, Klaver NS, van Gestel TJ and van de Klundert J (2020) Intention to use medical apps among older adults in the Netherlands: Cross-sectional study. *Journal of Medical Internet Research* 22(9): e18080.
4	Berkowsky RW, Sharit J and Czaja SJ (2017) Factors predicting decisions about technology adoption among older adults. *Innovation in Aging* 1(3): igy002.
5	Braun MT (2013) Obstacles to social networking website use among older adults. *Computers in Human Behavior* 29(3): 673–680.
6	Cajita MI, Hodgson NA, Lam KW, Yoo S and Han HR (2018) Facilitators of and barriers to mHealth adoption in older adults with heart failure. *Computers, Informatics, Nursing: CIN* 36(8): 376.
7	Chen K (2020) Why do older people love and hate assistive technology? – An emotional experience perspective. *Ergonomics* 63(12): 1463–1474.
8	Chen K and Lou VWQ (2020) Measuring senior technology acceptance: Development of a brief, 14-item scale. *Innovation in Aging* 4(3): igaa016.
9	Choudrie J and Vyas A (2014) Silver surfers adopting and using Facebook? A quantitative study of Hertfordshire, UK applied to organizational and social change. *Technological Forecasting and Social Change* 89: 293–305.
10	Chu L, Chen HW, Cheng PY, Ho P, Weng IT, Yang PL, Chien SE, Tu YC, Yang CC, Wang TM and Fung HH (2019) Identifying features that enhance older adults’ acceptance of robots: A mixed methods study. *Gerontology* 65(4): 441–450.
11	Chung J, Thompson HJ, Joe J, Hall A and Demiris G (2017) Examining Korean and Korean American older adults’ perceived acceptability of home-based monitoring technologies in the context of culture. *Informatics for Health and Social Care* 42(1): 61–76.
12	Cimperman M, Brenčič MM, Trkman P and Stanonik MD (2013) Older adults’ perceptions of home telehealth services. *Telemedicine and e-Health* 19(10): 786–790.
13	Courtney KL (2008) Privacy and senior willingness to adopt smart home information technology in residential care facilities. *Methods of Information in Medicine* 47(1): 76–81.
14	Dequanter S, Fobelets M, Steenhout I, Gagnon MP, Bourbonnais A, Rahimi S, Buyl R and Gorus E (2022) Determinants of technology adoption and continued use among cognitively impaired older adults: A qualitative study. *BMC Geriatrics* 22(1): 1–6.
15	Dermody G, Fritz R, Glass C, Dunham M and Whitehead L (2021) Factors influencing community-dwelling older adults’ readiness to adopt smart home technology: A qualitative exploratory study. *Journal of Advanced Nursing* 77(12): 4847–4861.
16	Fisher K and Easton K (2019) The meaning and value of digital technology adoption for older adults with sight loss: A mixed methods study. *Technology and Disability* 30(4): 177–184.
17	Ghorayeb A, Comber R and Gooberman-Hill R (2021) Older adults’ perspectives of smart home technology: Are we developing the technology that older people want? *International Journal of Human-Computer Studies* 147: 102571.
18	González A, Ramírez MP and Viadel V (2015) ICT learning by older adults and their attitudes toward computer use. *Current Gerontology and Geriatrics Research* 2015: 849308.
19	Greenhalgh T, Wherton J, Sugarhood P, Hinder S, Procter R and Stones R (2013) What matters to older people with assisted living needs? A phenomenological analysis of the use and non-use of telehealth and telecare. *Social Science & Medicine* 93: 86–94.
20	Haghzare S, Campos JL, Bak K and Mihailidis A (2021) Older adults’ acceptance of fully automated vehicles: Effects of exposure, driving style, age, and driving conditions. *Accident Analysis & Prevention* 150: 105919.
21	Harris MT and Rogers WA (2021) Developing a Healthcare Technology Acceptance Model (H-TAM) for older adults with hypertension. *Ageing & Society* 2: 1–21.
22	Heart T and Kalderon E (2013) Older adults: Are they ready to adopt health-related ICT? *International Journal of Medical Informatics* 82(11): e209–e231.
23	Herscovici A and Manor S (2022) Living in the digital periphery – Old people in rural Israel talk about information technology. *Rural Sociology* 87(1): 186–205.
24	Hong SI (2016) Community older adults’ attitude towards the use of assistive devices. *Asia Pacific Journal of Social Work and Development* 26(4): 217–230.
25	Huang H, Chen Z, Cao S, Xiao M, Xie L and Zhao Q (2021) Adoption intention and factors influencing the use of gerontechnology in Chinese community-dwelling older adults: A mixed-methods study. *Frontiers in Public Health* 9: 1302.
26	Ienca M, Schneble C, Kressig RW and Wangmo T (2021) Digital health interventions for healthy ageing: A qualitative user evaluation and ethical assessment. *BMC Geriatrics* 21(1): 1–10.
27	Jeng MY, Pai FY and Yeh TM (2022) Antecedents for older adults’ intention to use smart health wearable devices-technology anxiety as a moderator. *Behavioral Sciences* 12(4): 114.
28	Hwang YS (2021) Psychological factors that affect the acceptance and need for ICT services for older adults with chronic diseases. *Gerontechnology* 20(2): 1–11.
29	Kadylak T, Cotten SR and Fennell C (2021) Willingness to use automated vehicles: Results from a large and diverse sample of US older adults. *Gerontology and Geriatric Medicine* 7: 1–10.
30	Klaver NS, Van de Klundert J and Askari M (2021) Relationship between perceived risks of using mHealth applications and the intention to use them among older adults in the Netherlands: Cross-sectional study. *JMIR mHealth and uHealth* 9(8): e26845.
31	Lai CK, Chung JC, Leung NK, Wong JC and Mak DP (2010) A survey of older Hong Kong people’s perceptions of telecommunication technologies and telecare devices. *Journal of Telemedicine and Telecare* 16(8): 441–446.
32	Lesauskaitė V, Damulevičienė G, Knašienė J, Kazanavičius E, Liutkevičius A and Janavičiūtė A (2019) Older adults – Potential users of technologies. *Medicina* 55(6): 253.
33	Li J, Ma Q, Chan AH and Man S (2019) Health monitoring through wearable technologies for older adults: Smart wearables acceptance model. *Applied Ergonomics* 75: 162–169.
34	Lie ML, Lindsay S and Brittain K (2016) Technology and trust: Older people’s perspectives of a home monitoring system. *Ageing & Society* 36(7): 1501–1525.
35	Liu D, Liu A and Tu W (2020) The acceptance behavior of new media entertainment among older adults: Living arrangement as a mediator. *The International Journal of Aging and Human Development* 91(3): 274–298.
36	Louie WY, McColl D and Nejat G (2014) Acceptance and attitudes toward a human-like socially assistive robot by older adults. *Assistive Technology* 26(3): 140–150.
37	Lai CK, Chung JC, Leung NK, Wong JC and Mak DP (2015) A survey of older Hong Kong people’s perceptions of telecommunication technologies and telecare devices. *Journal of Telemedicine and Telecare* 16(8): 441–446.
38	Ma Q, Chan AH and Chen K (2016) Personal and other factors affecting acceptance of smartphone technology by older Chinese adults. *Applied Ergonomics* 54: 62–71.
39	Macedo IM (2017) Predicting the acceptance and use of information and communication technology by older adults: An empirical examination of the revised UTAUT2. *Computers in Human Behavior* 75: 935–948.
40	Mackenzie L and Clifford A (2020) Perceptions of older people in Ireland and Australia about the use of technology to address falls prevention. *Ageing & Society* 40(2): 369–388.
41	Mahmood N and Lee YA (2021) Factors influencing older adults’ acceptance of health monitoring smart clothing. *Family and Consumer Sciences Research Journal* 49(4): 376–392.
42	Martín-García AV, Redolat R and Pinazo-Hernandis S (2021) Factors influencing intention to technological use in older adults. The TAM model application. *Research on Aging* 44(7–8): 573–588.
43	Melenhorst AS, Rogers WA and Bouwhuis DG (2006) Older adults’ motivated choice for technological innovation: Evidence for benefit-driven selectivity. *Psychology and Aging* 21(1): 190–195.
44	Menéndez Álvarez-Dardet S, Lorence B and Pérez-Padilla J (2020) Older adults and ICT adoption: Analysis of the use and attitudes toward computers in elderly Spanish people. *Computers in Human Behavior* 110: 106377.
45	Mitzner TL, Boron JB, Fausset CB, Adams AE, Charness N, Czaja SJ, Dijkstra K, Fisk AD, Rogers WA and Sharit J (2010) Older adults talk technology: Technology usage and attitudes. *Computers in Human Behavior* 26(6): 1710–1721.
46	Nägle S and Schmidt L (2012) Computer acceptance of older adults. *Work* 41(Supp. 1): 3541–3548.
47	Nayak LU, Priest L and White AP (2010) An application of the technology acceptance model to the level of Internet usage by older adults. *Universal Access in the Information Society* 9(4): 367–374.
48	O’Brien J, Mason A, Cassarino M, Chan J and Setti A (2021) Older women’s experiences of a community-led walking programme using activity trackers. *International Journal of Environmental Research and Public Health* 18(18): 9818.
49	Outila M and Kiuru H (2020) ‘Picturephone in my home’: Actor-network theory and Foucauldian discourse analysis on Northern Finnish older adults starting to use a video conferencing service. *Journal of Technology in Human Services* 39(2): 163–192.
50	Pan J, Dong H and Bryan-Kinns N (2021) Perception and initial adoption of mobile health services of older adults in London: Mixed methods investigation. *JMIR Aging* 4(4): e30420.
51	Pan S and Jordan-Marsh M (2010) Internet use intention and adoption among Chinese older adults: From the expanded technology acceptance model perspective. *Computers in Human Behavior* 26(5): 1111–1119.
52	Peek ST, Luijkx KG, Rijnaard MD, Nieboer ME, Van Der Voort CS, Aarts S, Van Hoof J, Vrijhoef HJ and Wouters EJ (2016) Older adults’ reasons for using technology while aging in place. *Gerontology* 62(2): 226–237.
53	Peterson KF and Adams-Price C (2022) Fear of dependency and life-space mobility as predictors of attitudes toward assistive devices in older adults. *The International Journal of Aging and Human Development* 94(3): 273–289.
54	Pirhonen J, Lolich L, Tuominen K, Jolanki O and Timonen V (2020) “These devices have not been made for older people’s needs” – Older adults’ perceptions of digital technologies in Finland and Ireland. *Technology in Society* 62: 101287.
55	Rahman MM, Deb S, Strawderman L, Burch R and Smith B (2019) How the older population perceives self-driving vehicles. *Transportation Research Part F: Traffic Psychology and Behaviour* 65: 242–257.
56	Rasekaba TM, Pereira P, Rani GV, Johnson R, McKechnie R and Blackberry I (2022) Exploring telehealth readiness in a resource limited setting: Digital and Health Literacy among Older People in Rural India (DAHLIA). *Geriatrics* 7(2): 28.
57	Safarov N (2021) Personal experiences of digital public services access and use: Older migrants’ digital choices. *Technology in Society* 66: 101627.
58	Sánchez VG, Anker-Hansen C, Taylor I and Eilertsen G (2019) Older people’s attitudes and perspectives of welfare technology in Norway. *Journal of Multidisciplinary Healthcare* 12: 841–853.
59	Schehl B, Leukel J and Sugumaran V (2019) Understanding differentiated Internet use in older adults: A study of informational, social, and instrumental online activities. *Computers in Human Behavior* 97: 222–230.
60	Seifert A and Schelling HR (2015) Mobile use of the Internet using smartphones or tablets by Swiss people over 65 years. *Gerontechnology* 14(1): 57–62.
61	Selwyn N (2004) The information aged: A qualitative study of older adults’ use of information and communications technology. *Journal of Aging Studies* 18(4): 369–384.
62	Selwyn N, Gorard S, Furlong J and Madden L (2003) Older adults’ use of information and communications technology in everyday life. *Ageing & Society* 23(5): 561–582.
63	Sixsmith A, Horst BR, Simeonov D and Mihailidis A (2022) Older people’s use of digital technology during the COVID-19 pandemic. *Bulletin of Science, Technology & Society* 42(1–2): 19–24.
64	Smarr CA, Mitzner TL, Beer JM, Prakash A, Chen TL, Kemp CC and Rogers WA (2014) Domestic robots for older adults: Attitudes, preferences, and potential. *International Journal of Social Robotics* 6(2): 229–247.
65	Su J and Tong X (2021) Catching silver consumers in China: An integrated model of Chinese older adults’ use of social networking technology. *Asia Pacific Journal of Marketing and Logistics* 33(9): 1903–1917.
66	Talukder MS, Sorwar G, Bao Y, Ahmed JU and Palash MA (2020) Predicting antecedents of wearable healthcare technology acceptance by elderly: A combined SEM-Neural Network approach. *Technological Forecasting and Social Change* 150: 119793.
67	Teh PL, Lim WM, Ahmed PK, Chan AH, Loo JM, Cheong SN and Yap WJ (2017) Does power posing affect gerontechnology adoption among older adults? *Behaviour & Information Technology* 36(1): 33–42.
68	Thomas L, Little L, Briggs P, McInnes L, Jones E and Nicholson J (2013) Location tracking: Views from the older adult population. *Age and Ageing* 42(6): 758–763.
69	Tobis S, Piasek J, Cylkowska-Nowak M and Suwalska A (2022) Robots in eldercare: How does a real-world interaction with the machine influence the perceptions of older people? *Sensors* 22(5): 1717.
70	Tsai TH, Chang HT, Chen YJ and Chang YS (2017) Determinants of user acceptance of a specific social platform for older adults: An empirical examination of user interface characteristics and behavioral intention. *PLoS ONE* 12(8): e0180102.
71	Tsai TH, Lin WY, Chang YS, Chang PC and Lee MY (2020) Technology anxiety and resistance to change behavioral study of a wearable cardiac warming system using an extended TAM for older adults. *PLoS ONE* 15(1): e0227270.
72	Tural E, Lu D and Austin Cole D (2021) Safely and actively aging in place: Older adults’ attitudes and intentions toward smart home technologies. *Gerontology and Geriatric Medicine* 7: 1–15.
73	Tural E, Lu D and Cole DA (2020) Factors predicting older adults’ attitudes toward and intentions to use stair mobility assistive designs at home. *Preventive Medicine Reports* 18: 101082.
74	Vicente P and Lopes I (2016) Attitudes of older mobile phone users towards mobile phones. *Communications* 41(1): 71–86.
75	Wang L, Rau PL and Salvendy G (2011) Older adults’ acceptance of information technology. *Educational Gerontology* 37(12): 1081–1099.
76	Wang L, Chen J and Ju DY (2021) Factors contributing to Korean older adults’ acceptance of assistive social robots. *Electronics* 10(18): 2204.
77	Wildenbos GA, Jaspers MW, Schijven MP and Dusseljee-Peute LW (2019) Mobile health for older adult patients: Using an aging barriers framework to classify usability problems. *International Journal of Medical Informatics* 124: 68–77.
78	Wong CK, Yeung DY, Ho HC, Tse KP and Lam CY (2014) Chinese older adults’ Internet use for health information. *Journal of Applied Gerontology* 33(3): 316–335.
79	Wu YH, Wrobel J, Cornuet M, Kerhervé H, Damnée S and Rigaud AS (2014) Acceptance of an assistive robot in older adults: A mixed-method study of human–robot interaction over a 1 month period in the Living Lab setting. *Clinical Interventions in Aging* 9: 801–811.
80	Yang CC, Li CL, Yeh TF and Chang YC (2022) Assessing older adults’ intentions to use a smartphone: Using the meta–unified theory of the acceptance and use of technology. *International Journal of Environmental Research and Public Health* 19(9): 5403.
81	Young R, Willis E, Cameron G and Geana M (2014) “Willing but unwilling”: Attitudinal barriers to adoption of home-based health information technology among older adults. *Health Informatics Journal* 20(2): 127–135.
82	Zhao S, Yao Y and Ya N (2021) Adoption of mobile social media for learning among Chinese older adults in senior citizen colleges. *Educational Technology Research and Development* 69(6): 3413–3435.
83	Zhou J, Rau PL and Salvendy G (2014) Older adults’ use of smart phones: An investigation of the factors influencing the acceptance of new functions. *Behaviour & Information Technology* 33(6): 552–560.

### Data extraction and analysis

A data extraction form was used for collecting the information and main contents of the 83 articles. The structured information included the following: author, year of publication, title of research, source, abstract, country/region of research, participants (number of participants and inclusion criteria), theoretical model, methodology and types of technology involved.

The author perused all 83 articles and initially coded the sections of findings (results) and discussion. The focus of coding was on how researchers talked about older people, how they described older adults’ attitudes towards technology, the types of factors influencing attitudes and adoption, the reasons for these factors, and the conclusion and limitation of research. Informed by the framework of three categories of factors influencing technology adoption for all populations in the introduction above (i.e. personal characteristics, technology-related factors and social factors; the framework was also used in the work of Cajita et al. (2018) and Dequanter et al. (2022)), the initial codes were organised into these three first-level themes, with sub-themes under each of them.

## 3. Findings

### Overview

**Figure 1.**
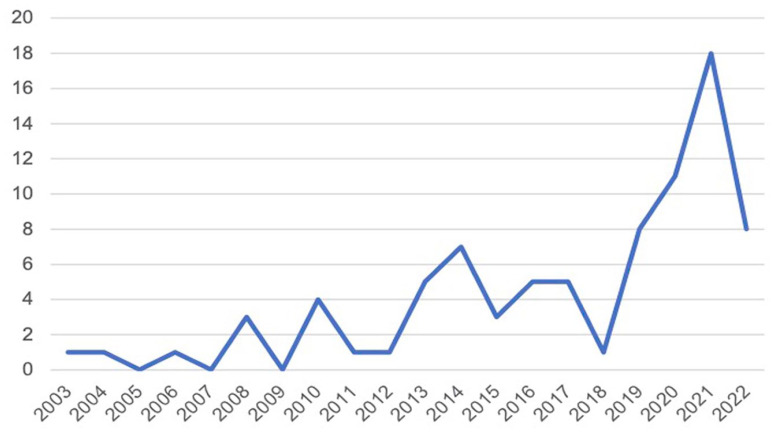
Number of articles published by year.

The 83 articles were published within 20 years (2003–2022) with a growth in the past 3 years (though research in 2022 was not fully covered) (Figure 1). While early researchers mostly focused on older people’s adoption of the Internet, mobile phones and other information and communications technologies (ICTs), literature in the past 10 years (*n* = 71) involved diverse technologies with more fine classifications, including assistive technologies, social networks, automated vehicles and robots.

**Figure 2.**
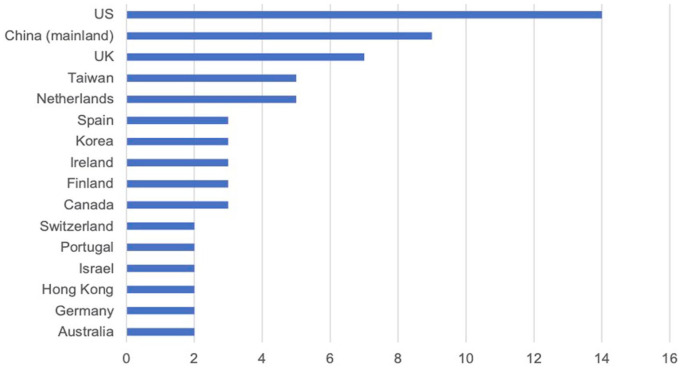
Main countries or regions included in research.

Likewise, the countries or regions covered by the research were also expanded over time. Before 2010, almost all relevant studies were observing Europe (especially the United Kingdom) and the United States; but in the past 10 years, 71 articles covered 25 countries or regions with increasing publications from East Asian and Eastern European countries. Researchers also conducted cross-cultural comparisons of older adults’ attitudes towards technologies (11, 54). Figure 2 shows the main countries or regions included in all studies.

More than half (*n* = 50) of the studies employed quantitative methods, such as launching a questionnaire survey on a large range of older people and measuring their attitudes with scales. Other research used qualitative methods like interviews (*n* = 24), focus groups (*n* = 15), and ethnography (including long-term observations and conversations, *n* = 3). Some studies (*n* = 23) used mixed methods to obtain more comprehensive data. The number of respondents ranged from 8 to 1923.

### Older people’s personal characteristics and their attitudes towards technology

#### Age

Many studies argued that compared with young and middle-aged people, older adults would encounter more barriers and have more negative attitudes towards technology. Some of them further demonstrated that older people are not a homogeneous group and subdivided the age groups among older people. They found that the willingness of older seniors to adopt technologies is significantly lower than that of younger seniors (4, 20, 31, 32, 38, 51, 59, 60, 62, 72, 73). Researchers attributed the reasons to the declining health status and conservatism with age, which was defined as sticking to old habits and refusing to accept changes (33, 54, 57, 77).

#### Gender

The gender differences were mentioned in 17 articles. Older men were more likely to use automated vehicles, computers, and smartphones (29, 46, 51, 55, 60, 78) and were active users of ICTs (44), while women used assistive devices more often (24). Researchers believed that these differences are associated with the social and cultural image of gender, and older adults might conceptualise the use of technology through a gendered lens. For example, older men’s preference for autonomous living and the consciousness of being ‘workers’ that they retain after retirement led to more desire for technologies for lifestyle management, and women, with the role of ‘wives and mothers’, tended to require technological services on which they could be dependent (28).

One-third of the studies had a high proportion of women participants (over 65% of all participants), but only two of them explained the reason: the participants were recruited from community centres, and women were more interested in activities happening there (paper 7, with 88% female participants); women retired earlier than men and enrolled more in the ‘senior citizen colleges’, which was the site of the research (paper 82, with 81% female participants). This may lead to insufficient data on older men and discrepancies with the actual situation.

#### Education and income

Higher educational background and income levels have a positive impact on technology adoption (1, 22, 29, 31, 33, 38, 44, 47, 56, 59, 62, 74). Research showed that older adults with higher levels of education and income have higher cost affordability and more positive attitudes towards using technology (38), and this effect is more pronounced for personal, complex and structured tools (59). However, paper 18 suggested that the effects of education and income should be correlated to mediating variables such as experiences or cognitive skills, which were also proven to be important factors positively influencing adoption (6, 28, 56, 82).

#### Other personal factors

In addition, a few studies pointed out the possible impact of health beliefs, willingness to learn and living arrangement on technology adoption. Health beliefs are primarily related to the use of health technology, referring to the observation that older people who have experienced diseases are more aware of the importance of health management (25, 50). A higher willingness to learn increases motives to use new technologies for promoting active ageing (6, 18, 49), while older adults who regard learning as a nuisance have a strong rejection of new devices (28). The impact of living arrangement is reflected in the acceptance of smart home technologies and assistive devices, as older adults with home ownership, greater living space and fewer people living at home have higher intention to use (53, 72).

### Technology-related factors that affect attitudes and adoption

#### Access and cost

Among the technology-related factors, the most fundamental ones are whether older people could get access to the technology and how they think of the cost. The two were often raised together in literature and are associated with the ‘grey digital divide’, which represents the digital accessibility issues of older people compared with young people (14, 23, 39, 44, 45, 47, 49, 57, 61, 72). The grey digital divide is further categorised into the ‘primary digital divide’ (indicating older people without access) and the ‘secondary digital divide’ (indicating older people who have access to technology but do not use it) (44). The primary digital divide generally stems from inequalities in economic and social conditions, as some older people are unable to install devices due to financial constraints (47, 50). Discussions of the secondary digital divide are about the reasons why older people actively choose not to use, which include lack of information, high cost, disinterest and lack of need (1, 19, 20, 22, 44, 47, 61, 62).

During the trial and use of technology, cost remains a common consideration for older people (6, 14–17, 28, 31, 35, 38, 42, 52, 58). Researchers established a link between cost and affordability, where older people with no income and no children are more sensitive to the price of technology and less likely to accept high-priced products (28, 35); conversely, for affordable technologies, consideration of cost is no longer a major influence (39). Older people tend to compare the benefits of the technology to the costs, in favour of the technologies with high perceived benefits, which is in accordance with the model of Selective Optimization With Compensation (SOC) (43, see also Baltes and Baltes, 1990). Some participants also expressed views on public policy and the commercialisation of technology when considering costs. For example, Korean participants seemed to regard the government’s ability to reduce the cost and increase accessibility as an ‘indicator of technological capabilities for developed countries’ (11).

#### Technology adoption models and their extensions

There are 44 articles citing different models to structure the main factors influencing older people’s adoption of technology, including TAM and its extension (TAM2, TAM3, *n* = 32) (Davis, 1989; Venkatesh and Bala, 2008; Venkatesh and Davis, 2000), UTAUT and its extension (UTAUT2, *n* = 23) (Venkatesh et al., 2003, 2012) and STAM (the senior technology acceptance model, *n* = 8) (Chen and Chan, 2014). Most of these articles constructed questionnaires based on the factors in the models to investigate the extent to which older people’s attitudes, behavioural intentions and use of technology are influenced by factors such as ‘perceived usefulness’ in TAM (‘performance expectancy’ in UTAUT), ‘perceived ease of use’ in TAM (‘effort expectancy’ in UTAUT), ‘hedonic motivation’ in UTAUT2 and ‘technology anxiety’ in STAM.

Perceived usefulness (or performance expectancy) is one of the most important of the technology-related factors, with 28 papers mentioning its notable impact on older people’s attitudes or intention to use the technology. This illustrates that successful technology adoption among older people is need-driven and they care deeply about whether the use of technology makes them feel comfortable and enhances the efficiency of everyday life (14, 42), whereas the absence of visible advantages, low measuring accuracy and the risk of privacy leakage (accidental disclosure of personal information) decreases the perceived usefulness of technology for older people (33, 50). The perceived usefulness of some products emerges from comparisons with their alternatives. When older people feel satisfied with the existing products, the relative advantages of new technology may fall short (e.g. robots versus computers (79); electronic health records versus paper records (81)).

The effect of perceived ease of use (or effort expectancy) was mentioned in 19 studies. In assessing the adoption of mobile health (mHealth) technologies, researchers noted that ease of use was mainly considered in terms of the clarity of language and navigation, text size, unambiguousness of icons or buttons and ease of clicking (50, 77). Two articles found that for wearable devices (e.g. smartwatches), perceived ease of use did not directly influence behavioural intentions, but was mediated by perceived usefulness (33, 71). The relatively low impact of perceived ease of use could be ascribed to the simple design of technology, with which older participants were already proficient in the early stage of use and thus were not aware of the effect of ‘ease of use’ (46, 65, 66).

Hedonic motivation is a component of UTAUT2, defined as ‘the fun or pleasure derived from using a technology’ (Venkatesh et al., 2012). The debate on this factor was largely grounded on ICTs (9, 28, 35, 39, 65, 74). On the one hand, the recreational functions of ICTs help older people to cure boredom and facilitate connections, fostering positive attitudes towards technology (28, 39); on the other hand, hedonic motivation also has a negative effect on technology adoption when older people do not want to spend too much time on entertainment for fear of the risk of addiction and consuming energy (65, 74).

While the applications of TAM and UTAUT are centred on workplace and organisational settings, Chen and Chan (2014) developed STAM to specifically measure older people’s acceptance of technology, adding the factors of technology anxiety and other contextual factors (to be presented in the next section). The role of technology anxiety was validated in 11 articles, referring to the feelings of worry and fear of making mistakes about equipment operation and information exposure.

#### Further development of the models

In addition to the factors that exist in the conceptual model and have been widely verified, several researchers have also proposed other factors to further extend the models, including perceived security and reliability, the appearance and texture of the technology, and compatibility.

The security and reliability of technology are divided into environmental security (the increasing or weakening of the security for the living environment) and data security (data misuse and privacy leakage). Older people’s perceived security for ambient intelligence arises from its assistance in monitoring intruders and emergencies (15); and for digital health technology, perceived safety is achieved through security enhancements such as alarms (26). The reduction of perceived environmental security is often due to excessive intrusion or the absence of feedback about the system (17, 26).

Older people’s consideration of data security is combined with the protection of privacy and was covered by 21 articles. Some older people placed a high value on their privacy, being conservative about who can access the data and fearful of misuse of data by commercial organisations or hackers (15, 17, 26, 50, 68, 79, 81). There were also older people who do not worry about privacy leakage, because they found ‘nothing to hide at their age’ (34, 41, 68). Paper 17 identified a disappearance of privacy concerns after the experience of smart home technology for a few months. This captures a balance between older people’s vision of maintaining safety and privacy or compromises in some cases. Most older participants are willing to sacrifice a portion of privacy in exchange for the benefit of safety, provided they could retain sufficient autonomy over the technology use (13, 26, 58).

Ten articles referred to the appearance and texture of the technology, especially pointing out that older people would prefer wearables with larger screen size (Apple Watch over the Fitbit) (26) and robots with humanlike appearance and demeanour (10, 36). For home monitoring and assistive devices, older people want them to have a generic appearance, passing quite unnoticed to avoid strong stigma associations and to create pleasant experiences (7, 24, 28, 53, 68, 73).

Compatibility of technologies includes compatibility with existing lifestyles (21, 50), compatibility with existing equipment and facilities (55) and compatibility with pre-configurations made by designers (49). Paper 33 suggested that compatibility has a significant positive impact on perceived ease of use, perceived usefulness and intention to use.

### The social context of technology adoption

#### The sociotechnical construction of ageing and the citizenship of older adults

According to TAM2, older people’s acceptance of technology is also influenced by subjective norm and image (whether the behaviour of adoption is seen by others as reasonable, is in line with the social expectations for older people and could enhance their social status) (Venkatesh and Davis, 2000). Paper 3, 21 and 51 identified a key role of subjective norm, as older people’s perceptions of healthcare technology were influenced by advice from family members, friends and health service providers, but paper 22 and 28 argued that subjective norm was not a significant determinant of older people’s use of ICTs. Paper 74 found in a 2012 survey in Portugal that only a small proportion of older respondents (less than one in five) would regard a mobile phone as a social status object.

Specifically, older people are likely to be constructed in sociotechnical systems as dependent and in need of care, but they challenge these constructions through aspirations of maintaining independence. In some cases, older adults value doing things that symbolise independence and would consider using assistive devices as a way to compensate for deficiencies (15, 19, 53, 57). The self-development and the transformation of their lives through technology may contribute to a certain sense of ownership and power subjectivities (49). In other cases, however, the adoption of technology (especially assistive technology) is seen as a sign of stigma and losing independence. Older people sometimes perceive technology as being for even older or more vulnerable people than themselves and would show an attitude of ‘being still independent’ by rejecting the technology (15, 40, 68, 79).

#### The settings of technology

For home technology with a fixed location, the setting of the technology is of importance. Studies show that older people are sensitive to the location of electronic devices and may hold negative attitudes towards technology if it is installed in an inappropriate environment that occupies or disrupts their lifestyle (13, 17, 52, 64). Successful adaptation of technology is often characterised by ‘bricolage’, in which new devices could co-exist or combine with legacy ones, rather than replace them (19).

For technologies used in broader settings, older people think about the outside and social circumstances. For self-driving cars, older people take into account road safety and weather conditions and agree that suburban environments should be more suitable for interacting with self-driving vehicles than urban or rural areas (52, 55). Paper 23 found that the concept of ‘smart village’ was developed in agglomerations of older people in Israel, where information technology (IT) was used as a means of compensating for the lack of agglomeration and providing services over distance. Older people recognised the contributions of technology to their lives but simultaneously regarded the transition to digital society as a threat to the pre-existing social environment. Their concerns about changes to the existing environment also include fears that technology will replace interpersonal interactions (28), professional caregivers (17, 30, 58) and face-to-face services (54, 57, 66).

#### The impact of social networks

The construction of older people’s technological identity also revolves around social networks of different breadths, including influence from family and peers, from professionals, from organisational training and support, as well as social and institutional influences. The first two items are formulated as ‘social influence’ in the UTAUT model while the latter two are classified as ‘facilitating conditions’ (Venkatesh et al., 2003). Five studies showed that social influence is significantly associated with older people’s intentions to adopt technology. Ten studies mentioned the factor of ‘facilitating conditions’, but paper 39 and 51 found limited effects of facilitating conditions on actual use, possibly because the role of organisational and social support was no longer apparent once older people became proficient in use. The four dimensions of social networks are detailed in the following.

Family and peers were the sources of social influence that appeared most frequently in the studies. On the one hand, the aspiration to be in touch with family and peers both motivates and repulses use of technology. Some older people use computers and other ICTs to communicate with (distant) family and provide reassurance to their children (23, 37, 52), while some others perceive ICTs as a distraction and believe that these technologies are posing a new form of alienation because people are always busy with phones when they stay together or tend to send messages instead of visiting in-person (23). On the other hand, in terms of actual encounters and use, older participants receive a wealth of help and support from family and peers (9, 12, 14–16, 22, 56, 62, 75). First, older people rarely purchase new computers independently but often acquire them through the process of recycling or redistribution from family members (61). Second, after older people have their own ICTs, their children or grandchildren will take on the role of ‘bricoleur’ to encourage, advise them to use ICTs and to intervene in their use as ‘gatekeepers’ (19, 37, 61). However, the process of seeking help is also accompanied by a consideration of whether it is safe and comfortable to disclose their ignorance. Older people would prefer natural influence from their partner (37) but sometimes they are reluctant to ask for help because they do not want their family members to access their data (12, 57). A situation of power reversal is found in the process, as young people’s role as teachers and impatience in tutoring would make older people feel disempowered (23, 49); but interestingly, due to more obvious intergenerational gaps, older people display great pride and pleasure when they receive help from their grandchildren (37).

The influence of professionals is mainly manifested in older people’s adoption and use of health technology. Doctors are reported as an essential source of information for the adoption of home telehealth services (HTS) and medical technologies (6, 12), with older people’s trust and respect for professionals (11). These supports are also crucial when older people lack confidence in using technology (40, 56).

In the early stages of adopting new technologies, communities and technical organisations provide training and support for older adults. Twelve studies demonstrated the responsibility and potential of communities and organisations to provide regular courses and assistance, but also pointed out the shortcomings of current services. Some older people are happy to attend these courses in the hope of adapting to new times (18), but some are not interested in training, automatically associating digital technologies with complexity and assuming that they are not able to grasp them through training (16). Five studies discussed the supportive role of institutions in offering product trials, marketing and advertising (1, 14, 38, 50, 52). Paper 1 and paper 79 addressed the necessity of technical organisations to provide maintenance and post-purchase services.

The support from the whole social system and institutions is included in a broader sense of facilitating conditions. Older participants identified the institutional support required in technology adoption, such as the desire for the government to develop public policies to improve the accessibility and centralise the management of technology (11); to offer financial subsidies or insurance for technology (24–26, 73) and to implement public education programmes and improve digital literacy (24). On the contrary, the forced deployment of some technologies also results in resistance and rejection by older adults. Sometimes the adoption of a technology is not really the choice of the user, but it is the growing popularity or the trend of the times that makes the use inevitable, like significantly more older adults over 75 reported using the Internet daily during the COVID-19 pandemic for online shopping and social activities (63). However, alongside the popularity of technology, older people are also fearful of the alienation and control associated with the information society (23, 54); are concerned about the transfer of responsibility for the services and the risk of taking on a consumer role after the traditional in-person services are all replaced by technologies (34, 49, 57); and feel anxious about new inequalities caused by the pursuit of economic efficiency (49, 54, 66).

#### Cultural factors

The use of technology is also an embodiment of users’ cultural values (19). Cultural factors influencing technology adoption are more often reported in the literature about Asian countries. For example, in South Korea, economic development has changed the filial expectation of older people to receive support from their children, which becomes a distinctive factor in older people’s acceptance of home-based monitoring technologies (11). In Israel, the post-modern culture of using digital technologies contradicts many norms and beliefs held by older people, who cling to familiar and reliable habits and take pride in being ‘old school’ (23). However, more studies identified their own limitations of contextual homogeneity and lack of focus on the cultural aspect (8, 22, 35, 38, 65).

## 4. Discussion

From the review of the 83 articles, it was found that older people’s attitudes towards the adoption and use of technology are influenced by their own characteristics (including age, gender, education and income), technology-related factors (such as access and cost, perceived usefulness and perceived ease of use) and social context (social construction of ageing, the setting of technology, social networks and cultural factors). According to the social shaping of technology, technology is an integrated ‘system’ consisting of economic, political and cultural aspects of society (MacKenzie and Wajcman, 1999: 17–18). The process of framing and constructing these factors is discussed in detail below.

### The construction of ‘older people’s identity’

First of all, the construction of older people’s identity could be observed in the way that researchers described older people’s interaction with technology, which shaped their choice to include older participants in their studies. Among all the studies, there is no consistent definition of ‘older people’. Only two papers explicitly explained the selection of participants, setting the inclusion criteria as people over 65, based on the World Health Organization’s definition of older people (11) or defined through the transition from work to retirement (59). ‘Old age’ is not solely a biological account, but is also socially and culturally constructed, and defined by the person’s ability to master the digitalising world (54). With the rapid increase in life expectancy in developed countries, the early 50s are still in their prime, healthy and working independently, and some researchers even believe that middle age begins at 60 (Bonnici, 2015). Defining older people as a group over 50 may not be appropriate in this context. However, in some of the most underdeveloped areas, the average life expectancy is still under 60 (United Nations, 2019). It is therefore not possible to generalise the age standard for all regions.

Furthermore, as research has proven that older people’s adoption of technology is related to their gender, education and income, the heterogeneity of older people needs to be considered. Existing studies have included a high proportion of female, high-income and well-educated participants, who might be relatively open-minded regarding technological innovations and are not representative of all older people (79; Neves and Amaro, 2012). This leads to a possible bias in the construction of the relationship between older people and technology. Segmenting the older generations and deconstructing the ageing process might be ways to take a fairer view of the relationship, for example, by looking at silver surfers (older and skilled users of digital technologies) and the impact of their application of technology in the workplace on later life (Olsson and Viscovi, 2020) and by incorporating more participants from less developed areas or with low incomes.

There are common instruments of making identities and making representations in the co-production of technology and society. The instrument of making identities refers to the redefinition or maintenance of identities under technological negotiation (Jasanoff, 2004: 39). Existing research has revealed how older people’s identities are co-produced with technology. On the one hand, technology solidifies the ‘stereotypes’ of older people about frailty and conservatism; on the other hand, older people challenge this perception of social identity by rejecting stigmatising technology. Making representations means that scientific and technological representations (including political and cultural implications) are made intelligible in practices (Jasanoff, 2004: 41). Representations co-produced with technology for older people are the devaluation of old skills and the lack of digital literacy, which has led to a suppression of power for them (Garvey and Miller, 2021: 110), but they also assert their traditional status and social order by refusing to be mentored.

### The construction of ‘technology’ and ‘the adoption of technology’

The construction of technology and the process of adoption are also evident in the literature. Most of the studies discussed what needs to be done to improve older people’s attitudes towards technology, suggesting that older people do not adopt technology because of certain ‘barriers’ and that withdrawal from technology is a ‘risk’. This discourse implies that they considered the adoption of technology to be an inevitable, correct and advantageous behaviour.

First, technology is not entirely good in practice. Although the development of digital technology has indeed provided convenience to people’s lives, making it faster to access information and bringing more choices in daily activities, studies also showed the drawbacks of technology (privacy leakage, digital fraud and stigma association) and concerns about misuse (e.g. 15, 19, 23, 24, 36). Some of these issues may be gradually improved with technological advances, but there are still inherent controversies that are hard to avoid, such as the lack of social contact due to excessive focus on technology (Vaportzis et al., 2017) and the question of how to deal with data (34).

Second, technology is not suitable for all people. Developers design technology with subjective assumptions and pre-configurations (Woolgar, 1990). They can not fully anticipate the needs of all users, especially older adults, with whom a generational gap exists. Not growing up with digital technology, many of the current older generations do not find technical terms as intuitive and easy to understand as younger people do; young people in turn may not understand why their older relatives need repeated training but are learning slowly (Miller et al., 2021: 161). One explanation for this is that the use of technology requires certain cognitive abilities, memory capacities and tacit knowledge, which are not owned by all people. Given the above, when researchers frame their studies in terms of social circumstances, they may need to take a more dialectical and objective perspective on technology adoption, to view ‘refusal to adopt’ and ‘stopping use’ as a general behaviour rather than wrongdoing, and to investigate the ‘reasons’ instead of ‘barriers’.

### The interaction of different factors

The various factors listed in the findings are not independent determinants affecting older people’s attitudes and adoption, and there are complex interactions between factors. These include (1) Influential effects: older people with higher self-efficacy have higher perceived ease of use and less technology anxiety (82); (2) Complementary effects: subsidies and reimbursements can alleviate concerns about the cost of technology (14, 73); (3) Contradictory effects: the contradiction between personal experiences (finding technology useless) and subjective norms (being expected to adopt); between ‘macro’ discourses of beneficial and empowering technology and limited usefulness of the technology in a ‘micro’ life perspective (61).

These interactions illustrate the limitations of quantitative research. First, it is difficult to fully untangle the effects of each variable. Second, older people’s attitudes towards technology sometimes can be complicated to describe and quantify (e.g. ‘sometimes easy, sometimes hard’ (Neves et al., 2015)). Third, even widely employed models of technology adoption are subject to the inclusion, intersection and refutation of multiple factors. And fourth, the accountability of models is strongly dependent on the participants and contexts, and the applicability of older models is questionable with changes in the nature of older people and technological progress. Therefore, it might be difficult to conduct reproducible quantitative studies with single-factor determinism and better to situate discussions within a specific population in a certain period.

### Older people’s role as co-designer

The expression of older people’s opinions in these studies plays a vital role in co-production. The current academic discourse is characterised by two divergent schools of thought: ones that base research on the one-way diffusion of technology to older people and cite technology (especially gerontechnology) as a means to ‘solve the problems of ageing’ and improve the lives of older adults (17, 25); the others counter the narrative that older people are only passive recipients of technology by emphasising the value of older people’s creative adoption and engagement in technology and that they take an active role in deviating from the scripts planned by technology companies (Loos et al., 2021). The concept of Responsible Research and Innovation (RRI) requires that societal actors and innovators take mutual responsibilities for the innovation process (Von Schomberg, 2011: 9), and further discussions on attitudes supported the potential for integrating older people’s perspectives into technology design and deployment, such as promoting older people’s collaboration within technology teams and bringing their experiences, knowledge, values and practical needs into the team (9).

However, there are many challenges in making the voices of older participants heard by other stakeholders (for example technology-related groups besides older users and non-users, such as technology developers and managers). This is because first, the image of older people as ‘passive users’ may still be embedded in the technology system and technology companies may not treat older people as equal partners. Second, the environment of engagement developed by technology companies may largely be about optimising brand image and achieving a higher level of user loyalty (65), without the purpose of collecting real feedback from older people. Third, in the broader political context, where public engagement is sometimes used to gain trust for a predetermined approach, those with power tend to engage the public in a structured way and to ignore suggestions that deviate from preconceptions, which in turn reinforces the incumbent power structures (Stilgoe et al., 2014). Fourth, there are also possible biases in the recruitment of older participants, such as a tendency to recruit healthy and active older people (e.g. Nielsen et al., 2018). Furthermore, a review by Fischer et al. (2020) suggests that the outcomes of older people’s involvement in technology design are still unknown and that engaging older participants does not necessarily yield better adoption and beneficial human-technology relationships.

Besides making identities and representations, Jasanoff (2004: 39–41) also talks about the co-production of discourses and institutions, which could further exert influence on technology. From the perspective of making discourses, existing reports and documents have usually positioned ageing as a social crisis, contributing to a moral high ground for the discourses of technology and innovation as the saviour (Neven and Peine, 2017), but the role of older people as active actors and as important resources to evaluate designs or guide ideation could be a rebuttal to the discourses of ‘ageing crisis’ (64). They construct a new body of knowledge and rhetoric through active adoption or rejection, engagement or refusal to engage. Yet the discursive choice made by scientific authorities in this regard remains unclear.

Older people’s appeals for institutional support and dissatisfaction with coercive digitisation exemplify preliminary attempts to join co-production in the politics and management of technology, especially for welfare technology and public service systems driven by government investment. They propose new institutional approaches, including striking a balance between the fast development of technology and the delivery of institutionalised training, drafting and enforcing relevant laws and guaranteeing the retention of traditional in-person services (54, 57). But again, the bond between older consumers and policymakers is difficult to forge, as it may require a fundamental shift in the distribution of power, with renegotiation, reformation and the development of new relational practices (Dunston et al., 2009).

### Research gap

There are limitations to the current studies. First, many studies acknowledge inadequate sample sizes or bias in the selection of participants. As a large number of participants were recruited from technology-related activities and some of the studies were conducted through online surveys, participants tend to be a relatively knowledgeable and interested group and could be outliers. Although such an approach is a convenient one for researchers to obtain robust data, we need to be concerned about the situation of public discourses and individual interests ‘being represented’ (Epstein, 2003: 177–182). In addition, though some research has included non-users, the investigations are still limited. Wyatt (2003) argues that non-users, former users and resisters are also important actors in shaping the technology, and it is necessary to distinguish between ‘have nots’ and ‘want nots’. Paper 55 compares the attitudes of ‘users of self-driving vehicles’ and ‘pedestrians’, but we still know little about how non-users or marginal users are affected by and act on technologies.

Second, sample articles are mostly about a single survey or comparative studies conducted before and after training or trials, while there are very few longitudinal studies. Lam and Lee (2006) point out the importance of longitudinal studies in disentangling complex cognitive constructs and understanding the duration of influences, but these are not elaborated in the existing research. Paper 22 and 62 suggest that older people’s major difficulties with adoption may be the physical limitations of old age and will not be eliminated by generation. It is therefore worth examining whether such age-related differences in technology attitudes will persist over time. Considering also that lockdowns during the COVID-19 pandemic may have created greater demand for digital technologies and remote services (50), more research is needed to explore whether this demand will influence the long-term use by older adults.

Third, there are more issues to be worked out regarding older people’s attitudes towards technology. For example, how do attitudes matter? We have taken a first glimpse at which factors influence older people’s perceptions of technology; if these factors are moderated, can technology be better tailored to the needs of older people? For instance, how might the security functions and privacy protection for monitoring technology be balanced (26)? For gerontechnology, how could age-appropriate design avoid stigmatisation? To answer these questions, there needs to be more interaction between technical staff and older people, allowing the attitudes of older people to be translated into the design of technology.

Some older people expressed the opinion that technology is not useful in their personal lives and preferred to use traditional services, which contradicts the expectation of universal adoption of technology (61). Such expectations may come from official reports and mass media to advocate for the public to become ‘ideal digital citizens’, connecting the use of technology with civic responsibilities (Schou and Hjelholt, 2019). We should consider whether these discourses overstate the advantages of technology to the detriment of some older people’s interests. Is it inevitable that new technologies will replace the old, or could old services remain available to people who prefer them? Jasanoff’s work on ‘sociotechnical imaginaries’ invites researchers to reflect on a collective vision of future life with the advances of science and technology (Jasanoff and Kim, 2015: 4–5). As older people are important members of the social community, there need to be more careful evaluations of embedding the future of the ‘ageing society’ in technological arrangements and the social deployment of technology.

## 5. Conclusion

This article reviews the literature on older people’s attitudes towards emerging technologies over the last two decades and finds that these attitudes are influenced by personal characteristics, technology-related factors and contextual factors. It analyses how researchers constructed these factors, such as in ways of emphasising the complex interplay of different factors and assigning different roles to older people as technology recipients, actors and co-designers. The limitations of current studies are also identified.

We argue that it is necessary to pay more attention to older people’s perceptions of technology and engage them in the design of technology, even if these tasks are arduous. For the long-term relationship between older people and technology, placing older people (both users and non-users) at the centre of research and complementing underrepresented groups of older people would allow for a more delicate balance between technological and social development, in order to achieve the goal of ‘responsible research and innovation’ (Wilkowska et al., 2018).
